# Adherence to a lifestyle intervention – just a question of self-efficacy? Analysis of the AgeWell.de-intervention against cognitive decline

**DOI:** 10.1186/s13195-024-01499-4

**Published:** 2024-06-22

**Authors:** Felix G. Wittmann, Alexander Pabst, Andrea Zülke, Melanie Luppa, Anke Oey, Melanie Boekholt, Solveig Weise, Thomas Fankhänel, Robert P. Kosilek, Christian Brettschneider, Juliane Döhring, Laura Lunden, Birgitt Wiese, Wolfgang Hoffmann, Thomas Frese, Jochen Gensichen, Hans-Helmut König, Hanna Kaduszkiewicz, Jochen René Thyrian, Steffi G. Riedel-Heller

**Affiliations:** 1 Institute of Social Medicine, Occupational Health and Public Health (ISAP), Medical Faculty, University of Leipzig, Phillip-Rosenthal-Str. 55, 04103 Leipzig, Germany; 2 Institute for General Practice, Work Group Medical Statistics and IT-Infrastructure, Hannover Medical School, Hannover, Germany; 3 Institute for Community Medicine, University Medicine Greifswald (UMG), Greifswald, Germany; 4 Institute of General Practice and Family Medicine, Martin-Luther-University Halle-Wittenberg, Halle, Saale, Germany; 5 Institute of General Practice and Family Medicine, University Hospital, LMU Munich, Munich, Germany; 6 Department of Health Economics and Health Service Research, University Medical Centre Hamburg-Eppendorf, Hamburg, Germany; 7 Institute of General Practice, University of Kiel, Kiel, Germany; 8 German Centre for Neurodegenerative Diseases (DZNE), Site Rostock/ Greifswald, Greifswald, Germany; 9 Faculty V: School of Life Sciences, University of Siegen, Siegen, Germany

**Keywords:** Dementia, Prevention, Lifestyle, Intervention, Adherence, Predictors

## Abstract

**Background:**

Aim of this study was to detect predictors of better adherence to the AgeWell.de-intervention, a two-year randomized multi-domain lifestyle intervention against cognitive decline.

**Methods:**

Data of 317 intervention group-participants comprising a risk group for dementia (Cardiovascular Risk Factors, Ageing and Dementia (CAIDE) score of  ≥ 9; mean age 68.9 years, 49.5% women) from the AgeWell.de intervention study were analysed. Regression models with four blocks of predictors (sociodemographic, cognitive and psychosocial, lifestyle factors and chronic conditions) were run on adherence to the components of nutrition, enhancement of social and physical activity and cognitive training. Adherence to each component was operationalised by assessing the degree of goal achievement per component at up to seven time points during the intervention period, measured using a 5-point Likert scale (mean score of goal achievement).

**Results:**

Increasing age was negatively associated with adherence, while higher education positively predicted adherence. Participants with better mental state (Montreal Cognitive Assessment (MoCA)-score > 25) at baseline and higher self-efficacy adhered better. Diabetes and cardiovascular conditions were not associated with adherence, whereas smoking negatively affected adherence. Highest education and quitting smoking in the past were the only predictors associated with all four intervention components.

**Conclusion:**

Results identified predictors for better and worse adherence. Particularly self-efficacy seems to be of considerable influence on adherence. This should be considered when designing future intervention trials.

**Trial registration:**

German Clinical Trials Register (ref. number: DRKS00013555).

## Introduction

The prevalence of dementia is increasing and is expected to triple in the next 30 years [1]. No curative treatment is available yet. Drug therapies promising to intervene in the mechanism that underlies dementia are not commonly available so far [2]. Meanwhile, research is focusing on risk reduction and prevention of dementia [3]. According to the Lancet Commission on dementia prevention, intervention, and care, a set of twelve modifiable risk factors are described to account for up to 40% of the risk for dementia on a population level [1]. These factors are lower education, hearing impairment, hypertension, obesity, diabetes, brain injury, smoking, excessive alcohol consumption, physical inactivity, depression, low social contact and air pollution [1]. A recent study calculated the potential to prevent cognitive decline for Germany. The results indicate a potential reduction of dementia prevalence in the next ten years of 138,000 cases if the prevalence of risk factors might be reduced by 15% [4]. These numbers underline the importance of conducting a more comprehensive and detailed examination of risk mitigation and preventive interventions.

In the light of modifiable risk factors, a promising approach to reduce the risk of cognitive decline thus lies in lifestyle interventions by targeting those risk factors. A first trial addressing several factors to preserve cognitive function was the Finnish Geriatric Intervention Study to Prevent Cognitive Impairment and Disability (FINGER), which showed small intervention effects on global cognition [5]. The idea of FINGER was subsequently adopted and adjusted to different economic, regional or cultural environments in other countries [6]. In Germany, the first multi-domain intervention trial was the AgeWell.de-trial [7]. The intervention included nutritional counseling, physical activity enhancement, cognitive training, optimization of medication, enhancement of social activity and intervention for bereavement, grief & depressive symptoms. Although the intervention did not reduce cognitive decline more effectively compared to the control group [8], the health-related quality of life was improved and depressiveness in women was reduced [9].

However, better adherence to the components nutrition and enhancement of social activity in AgeWell.de was linked to better cognitive performance at follow-up [8]. Hence, a key factor which may influence the effectiveness and thus a successful implementation of an intervention might be the adherence of recipients to the intervention components [10]. The participants of the intervention group were personally instructed by study nurses at beginning of the study in their homes, where individuals conducted the intervention under self-guidance. However, they were motivated by regular calls of the study nurses and weekly reflection notes. Furthermore, individual component goals were set at the beginning of the intervention. The approach chosen in AgeWell is different from other interventions. On one hand, participants were less supervised, on the other hand, the intervention may be seen as more closer to the participants everyday life. Thus, compared to the intervention of the FINGER study (which differs through closer supervision and conducting the intervention in the study centers [5]), the implementation of the AgeWell.de intervention was more self-reliant and home-adapted. Therefore, adherence is assumed to be of special relevance to the effectiveness of the AgeWell.de-intervention. Furthermore, a central result of previous intervention research against cognitive decline suggests that interventions should be tailored individually to the recipients as best as possible [3]. The research question we followed in this study was therefore: what predicted a better adherence to the intervention components in the AgeWell.de-study?

## Methods

### Study design & participants

Analyses were run using data of the AgeWell.de-study, a multi-domain cluster-randomized intervention over two years to preserve cognitive function in older adults (60–77 years old). Inclusion criteria was an age between 60–77 years and an increased risk for dementia (≥ 9 points in the CAIDE Score [11]). The CAIDE score contains information about age education, gender, blood pressure, body mass index, cholesterol and physical activity and is easy to assess by the GPs. Exclusion criteria were factors influencing the ability to safely participate in the intervention, participating in another intervention trial or diagnosis of dementia. Recruitment took place at five study sites (Greifswald, Kiel, Leipzig, Halle and Munich). Recruitment and initial screening were done in cooperation with GP practices. GP practices were randomized for recruitment of either intervention or control group participants by the data management center of the Institute for General Practice from the Hannover Medical School. Study design, recruitment and baseline data have been previously described in detail [7, 12]. Since no component was implemented for control group, no adherence data were surveyed for the control group which is why we only used participants of the intervention group.

The intervention was composed of six intervention components: (1) optimization of nutrition, a counselling subject to the guidelines of the German Nutrition Society; (2) enhancement of physical activity composed of a training program of aerobic training, muscle-strengthening activity and activity/balance exercise—a pedometer was provided to the study participants; (3) counselling on increasing social activity planned individually with the participants and accompanied with information about social activity, isolation and importance regarding risk of dementia; (4) cognitive training with a tablet computer and training software (NeuroNation ©) accompanied by information on risk and strategies and, at the least, (5) counselling on interventions of bereavement, grief and depressive symptoms and (6) a medication check with – in case of need – suggestions for optimization of medication. Study nurses introduced the intervention components face-to-face at the beginning of the intervention during home visits. In addition, participants received written information about nutritional counselling, physical, cognitive and social activity, information about cardiovascular system diseases and depression, grief and bereavement. In addition, goals were defined individually between the study nurse and the participants for social, cognitive and physical activity as well as the nutritional component at baseline. After 2, 4, 8, 16 and 20 months, study nurses were in contact with the participants by phone, one interim session was held face-by-face after 12 months. In every session, telephonic and face-by-face, the actual adherence to the goals were evaluated by the study nurse, respectively. The control group received treatment as usual by their general practitioner, in addition to written information on the intervention components described.

### Outcomes

The underlying question of adherence was the extent to which the recipient of the intervention was able to reach the goal of the intervention components and was answered by the study nurses following a discussion with the study participant, respectively. Adherence was surveyed for the components: nutrition, enhancement of physical and social activity and cognitive training. The other components were implemented individually. The corresponding goals were set individually with participants at the beginning of the intervention in a motivational interview setting with the study nurse. In detail, adherence to the intervention components enhancement of physical and social activity, cognitive training and nutrition were each surveyed by a question on a 5-point Likert scale (range 0–4, higher score indicating better adherence in the component) at seven time points (six monitoring sessions and one in-person interim evaluation throughout the course of the intervention). An adherence score was finally calculated of singular adherence data divided by seven for the time points as a mean score of adhering to the four components (range _low–high_ 0–4), respectively. The interviews at baseline and follow-up were conducted face-to-face, the interim sessions in between by telephone calls.

### Predictors

Four blocks of predictors of adherence were defined according to previous studies [10, 13, 14]. A first block contained sociodemographic variables including sex, age in years and education, operationalized according to the Comparative Analysis of Social Mobility in Industrial Nations scale (CASMIN [15]) with low education as reference. Cognitive and psychosocial factors were composed of, first, mild cognitive impairment (MCI) operationalized with the Montreal Cognitive Assessment [16] and a cut-off of 25/26 points [17]. Second, depressive symptoms were surveyed with the Geriatric Depression Scale (GDS, range_low-high_ 0–15) [18], and third, we used the 10-item Scale for General Self-Efficacy (SWE, total range_low-high_ 0–40 [19]) to assess self-efficacy. A third block of lifestyle factors consisted of smoking (never smoked as reference category; former smoker; current smoker), alcohol (high intake according to the European guideline of 14 units/week [20]), social inclusion using the Lubben Social Network Scale (LSNS, range_low-high_ 0–30) [21]; BMI and physical activity, used as dichotomous variable for at least 2 times 30 min per week vs. less physical activity. Finally, chronic conditions were analysed including diabetes, high blood pressure, history of stroke and heart diseases (cardiac arrhythmia or heart failure), all based on medical examination of the General Practitioner (GP). Except for education and smoking, we standardized all variables by computing z-scores for better comparison.

### Statistical analysis

Four generalized linear regression models (GLM) were used to analyse the effect of the predictors on adherence to the four intervention components, respectively. The adherence to the four components was used for each continuously without further categorisation. Since the adherence to the components were lightly left skewed, we controlled for the contribution within the GLM options. All models were calculated with the same core set of predictors and robust variance estimators, adjusting for variable types and distributions and finally accounting for clustering of participants in GP practices. The analyses were performed using STATA, Version 16(StataCorp, College Station, TX, USA).

## Results

### Participant characteristics

Of the 378 participants within the intervention group, data of 317 participants were complete for all predictors and for the adherence components. Baseline characteristics are presented in Table 1. Participants were aged between 60–77 years with a mean age of the analysed sample of 68.9 years, of which 49.5% (n = 157) were women.

**Table 1:** Baseline characteristics and adherence to the intervention components of the analyzed sample (*n* = 317)

Variable	Mean	SD/%
*Sociodemographic*		
Age (in years)	68.94	4.90
Women, (n)	157	49.5%
Education		
Low, (n)	80	25.2%
Intermediate, (n)	160	50.5%
High, (n)	77	24.3%
*Cognitive and psychosocial*		
MoCA < 26^a^, (n)	185	58.4%
Depressive symptoms^b^	1.45	1.85
Self-efficacy^c^	32.75	4.43
*Lifestyle*		
Smoking		
Never smoked, (n)	133	42.0%
Former smoker, (n)	142	44.8%
Current smoker, (n)	42	13.3%
Alcohol (high intake)^d^	83	26.2
Social Inclusion^e^	17.42	5.55
Body mass index	31.00	5.26
Physical active^f^, (n)	169	53.3%
*Chronic conditions*		
Diabetes, (n)	119	37.5%
Hypertension, (n)	285	89.9%
History of stroke, (n)	21	6.6%
Heart disease, (n)	95	30.0%
*Adherence to the components (range*_*low-high*_* 0–4)*		
Adherence to intervention component “nutrition”	2.78	0.70
Adherence to intervention component “physical activity”	2.64	0.82
Adherence to intervention component “social activity”	2.82	0.74
Adherence to intervention component “cognitive training”	2.92	0.76

^a^MoCA < 26 indicating MCI

^b^GDS, range (low–high) 0–15

^c^SWE-scale, range (low–high) 0–40

^d^Alcohol >  = 14 units / week according to European guideline

^e^Lubben Social Network Scale, range (low–high) 0–30

^f^Physical activity = at least twice weekly for minimum 30 min

Mean adherence was lowest for physical activity with 2.64 points, and highest for cognitive training with a mean of 2.92.

### Predictors of adherence

Adherence to the intervention component nutrition and enhancement to both social and physical activity decreased with age (Table 2). No effect of age was found for adherence to the component cognitive training. The difference between women and men had no effect on adherence except for the component cognitive training, where the adherence was higher among female participants. Higher education was positively associated with adherence for all components. However, participants with intermediate education did not differ regarding adherence to the component cognitive training from participants with lower education.

**Table 2:** Multiple linear regressions of predictors on adherence of intervention components of AgeWell.de (*n* = 317)

Characteristics	*Nutrition*		*Physical activity*		*Social activity*		*Cognitive training*	
	*Coef. (95% CI)*	*p*	*Coef. (95% CI)*	*p*	*Coef. (95% CI)*	*p*	*Coef. (95% CI)*	*p*
*Sociodemographic*								
Age	-0.077 (-0.134; -0.030)	**0.008**	-0.083 (-0.155; -0.010)	**0.026**	-0.083 (-0.147; -0.020)	**0.010**	-0.058 (-0.138; 0.021)	0.150
Sex (female)	0.056 (-0.024; 0.136)	0.170	-0.023 (-0.100; 0.054)	0.530	0.062 (-0.023; 0.148)	0.149	0.123 (0.022; 0.224)	**0.017**
Education								
low (ref.)								
intermediate	0.264 (0.113; 0.416)	**0.001**	0.323 (0.124; 0.501)	**0.001**	0.260 (0.113; 0.405)	**0.000**	0.151 (-0.016; 0.317)	0.077
high	0.466 (0.271; 0.662)	**0.000**	0.305 (0.135; 0.475)	**0.000**	0.468 (0.280; 0.660)	**0.000**	0.352 (0.142; 0.561)	**0.001**
*Cognitive and psychosocial*								
MoCA ≥ 26^a^	0.095 (0.014; 0.176)	**0.021**	0.063 (-0.030; 0.157)	0.185	0.075 (0.002; 0.149)	**0.043**	0.110 (0.024; 0.194)	**0.012**
Depressive symptoms^b^	-0.029 (-0.102; 0.044)	0.435	-0.100 (-0.176; -0.023)	**0.010**	-0.115 (-0.190; -0.039)	**0.003**	-0.072 (-0.181; 0.037)	0.193
Self-efficacy^c^	0.116 (0.034; 0.198)	**0.006**	0.130 (0.022; 0.236)	**0.018**	0.132 (0.038; 0.226)	**0.006**	0.059 (-0.020; 0.138)	0.143
*Lifestyle*								
Smoking								
Never smoked (ref.)								
Former smoker	-0.207 (-0.358; -0.056)	**0.007**	-0.193 (-0.360; -0.030)	**0.020**	-0.222 (-0.344; -0.100)	**0.000**	-0.211 (-0.343; -0.079)	**0.002**
Current smoker	-0.271 (-0.486; -0.055)	**0.014**	-0.355 (-0.620; -0.099)	**0.007**	-0.194 (-0.392; -0.004)	0.055	-0.403 (-0.673; -0.134)	**0.003**
Alcohol (high intake)^d^	-0.039 (-0.108; 0.030)	0.269	0.029 (-0.053; 0.111)	0.485	0.022 (-0.031; 0.076)	0.413	-0.057 (-0.131; 0.017)	0.134
Social support^e^	-0.008 (-0.081; 0.066)	0.839	0.098 (0.028; 0.168)	**0.006**	0.133 (0.053; 0.215)	**0.001**	0.006 (-0.075; 0.087)	0.885
Body mass index	-0.106 (-0.183; -0.029)	**0.007**	-0.134 (-0.221; -0.047)	**0.003**	-0.028 (-0.123; 0.067)	0.563	-0.014 (-0.101; 0.074)	0.754
Physical active^f^	0.049 (-0.011; 0.111)	0.110	0.235 (0.157; 0.312)	**0.000**	0.042 (-0.019; 0.103)	0.175	0.025 (-0.052; 0.102)	0.525
*Chronic conditions*								
Diabetes	0.017 (-0.052; 0.086)	0.624	-0.031 (-0.115; 0.054)	0.477	-0.060 (-0.076; 0.050)	0.690	-0.018 (-0.100; 0.061)	0.662
Hypertension	0.032 (-0.039; 0.102)	0.378	-0.002 (-0.080; 0.076)	0.959	-0.060 (-0.143; 0.024)	0.162	0.070 (-0.040; 0.181)	0.212
History of stroke	-0.013 (-0.111; 0.084)	0.789	-0.042 (-0.139; 0.054)	0.389	-0.032 (-0.101; 0.034)	0.334	-0.043 (-0.118; 0.033)	0.270
Heart disease	0.009 (-0.066; 0.083)	0.820	0.020 (-0.053; 0.093)	0.595	0.017 (-0.054; 0.087)	0.643	0.093 (0.010; 0.177)	**0.029**
* R*^*2*^		0.2044		0.3263		0.2805		0.1662

*CI* Confidence interval

^a^MoCA < 26 indicating MCI

^b^GDS, range (low–high) 0–15

^c^SWE-scale, range (low–high) 0–40

^d^Alcohol >  = 14 units / week according to European guideline

^e^Lubben Social Network Scale, range (low–high) 0–30

^f^Physical activity = at least twice weekly for minimum 30 min; all variables were z-standardized except of education and smoking

Participants with a MoCA score ≥ 26, indicating unimpaired mental state, showed higher adherence to the components nutrition, enhancement of social activity and cognitive training. Depressive symptoms were negatively associated with adherence to the component enhancement of physical and social activity but were not related to adherence to the components nutrition and cognitive training. With increasing self-efficacy, adherence to all components increased, except for adherence to the component cognitive training.

Former smokers showed less adherence to all components than participants who never smoked. The negative association was even stronger for participants who reported currently smoking. No association was found between the amount of current alcohol intake and adherence to the intervention components. Higher perceived social inclusion at baseline was associated with better adherence to the components physical and social activity.

Differences in diagnoses of chronic conditions like diabetes and cardiovascular diseases showed no measurable effect on adherence to the components, with one exception. Participants with heart disease showed better adherence to the component cognitive training.

The explained variance of the four components differed. While only 17% of the adherence to the component cognitive training could be explained with our set of predictors, we were able to cover 28% of the variance of better adherence to the component enhancement of social activity and nearly 33% of better adherence to the component enhancement of physical activity.

## Discussion

The aim of this study was to detect predictors of adherence to components of the AgeWell.de multi-domain lifestyle intervention against cognitive decline. We identified factors supporting better adherence, such as higher education, better cognitive status and higher levels of self-efficacy, but also found factors that hindered better adherence. Those were higher age, current or former smoking, depressive symptoms and a higher body mass index.

Very much in line with previous research is a negative association between age and better adherence [10, 13, 22]. In this study, this was found for nutrition and enhancement of social as well as physical activity. No association between age and adherence to cognitive training was found. Moreover, sex did not predict adherence, except for one component: Women adhered better to cognitive training than men.

The results of this study further showed that the higher the education, the better the adherence to all components. The link between education and health behaviour has been known for a long time [23], with known reasons such as family background, knowledge and cognitive ability, and also the social network of a person [24]. The intervention components were the same for all participants, and the components were accompanied with face-to-face instructions and extensive information material. Accordingly, all participants had the same level of information. Nevertheless, the access to the intervention components might have been unequally distributed between different education levels, for example regarding social activities or even more so healthier nutrition due to personal resources. This result reflect those of Geigl et al. (2022) who also found that socioeconomic status was negatively associated with dietary risk behaviour, supporting the need of a comprehensive understanding of the effect of social factors and nutrition in older adults [25]. Another possible explanation for the association between education and adherence bases on the theory of goal setting and goal orientation. According to that, education can be seen as moderator of effective goal setting. Thus, goal achievement, in the case of the study in the form of the appropriate operationalisation of adherence, is found partly to be explained by better competence of a more realistic goal setting and respective goal setting effectiveness, which is found more distinctly in persons with higher education. Reasons therefore are, among others, the competence to better assess one’s own abilities and also to assess the chance to achieve specified goals [26].

Another important finding is that both cognitive and psychosocial factors were significant predictors of adherence. Participants with unimpaired mental state (MoCA-score ≥ 26) performed better in all components, except for physical activity. An evident explanation is the positive relationship between executive functioning and motivation of adherence [27]. Executive functions are further defined in that they “(…) refer to a family of top-down mental processes needed when you have to concentrate and pay attention, (…) or relying on instinct or intuition would be ill-advised, insufficient, or impossible” [28]. This might support our result, since mild cognitive impairment is accompanied with lower executive functioning [27], and, in our study, is also associated with lower adherence to three components.

Depressive symptoms hindered adherence to the components enhancement of physical and social activity. The result is in line with findings of the FINGER study regarding social activities but differs regarding healthy diet and cognitive activities. This may be due to differences in the components. Nevertheless, this is a compelling finding, also because no respective effect on nutrition and cognitive training was found in AgeWell. Less physical and social activity is known to be associated with depressive symptoms. However, this result points out a reciprocal relationship between depressive symptoms and each social and physical activity [29]. Both are reported to decrease with depressive symptoms, while they are negatively related in the opposite direction as a way to reduce depressive symptoms. More-in-depth analysis of the effect of adherence of particular components on the secondary outcome of depressive symptoms might confirm this association. These findings further support the idea of better-targeted intervention trials. An implication of this result is that specific persons could benefit from even more individualized or group/need-oriented support or different goals, like persons with depressive symptoms.

Not unexpected is the positive association between self-efficacy and better adherence. Self-efficacy has long been known as one of the key factors for health behaviour, and even so for health behaviour change. Furthermore, the result is in line with previous research about self-efficacy and interventions [30–32]In an exploratory analysis, Neuvonen and colleagues already reported the relevance of psychosocial determinants for adherence such as depressive symptoms, hopelessness and dissatisfaction [33]. Nevertheless, self-efficacy has not been researched much with respect to dementia prevention yet. However, it is encouraging to see this finding in line with that found by Bruinsma et al. in a qualitative study about the perspectives on ligestyle-related behaviour change for dementia risk, reporting negative self-image and low behavioural control in relation to doubts towards implementing sustainable behaviour change [32]. A question that needs more investigation against the background of the goal to obtain better targeted and more effective interventions, and supported by literature and the results of the present study, is how to enhance self-efficacy in lifestyle trials. A theory-based approach could be an implementation of self-management factors into interventions. A study of a very different field (haemodialysis) to enhance quality of life, self-care and self-care behaviour showed positive effects of a self-management program on self-efficacy [34]. Furthermore, Dishman and colleagues found a mediating effect of self-management strategies for the relation between self-efficacy and physical activity [35]. Indeed, we included self-monitoring in the matter of weekly self-reflection notices. However, some other features to enhance self-efficacy in an intervention study could be among others providing regular structured feedback on the performance or planned social support as reported in a meta-analysis focusing on dietary interventions [36].

A very special role belongs to the adherence to cognitive training, which was not, as the other components were, associated with age, intermediate vs. low education, depressive symptoms and self-efficacy as well as with BMI-score and physical activity. Compared to the other components, the adherence to cognitive training was the highest. However, the variance we were able to explain the lowest. Adherence to cognitive training was analysed in detail within the FINGER study, where an effect of age was also not found. Also in contrast to the results in FINGER, our study showed that sex had an effect on adherence to cognitive training. FINGER investigated previous computer use, being married or cohabitating, better memory and positive study expectations and showed positive association with cognitive training [37]. While we did not assess information about previous computer use, our result might be consistent with FINGER regarding better adherence to cognitive training with better memory (in our study operationalised with MoCA-score cut off 25/26). A study focusing on a computer-based training program more deeply analysed determinants of adherence regarding MCI. They reported lower adherence to the program for participants with MCI, due to executive functioning in attention, working memory or cognitive flexibility [38]. Taken together, the results, nevertheless, underline the need for higher priority and other consideration of people with MCI to further maintain cognitive ability.

### Strengths and limitations

This study has several strengths. First, AgeWell.de used individual goal setting, which further allowed us to analyse adherence to individually set goals. Second, we analysed participants of a group in need, whereof, among others, 87.5% had hypertension, 40% diabetes and 55% were overweight. However, this study also has some limitations. First, adherence to the intervention components was measured as the mean of seven questions of how well participants reached their initially individually set goals. In other studies, adherence was operationalised by frequencies of participation in certain intervention activities. This must be taken into account when comparing the results to other adherence studies. For the other, we cannot totally exclude an effect of motivational interviewing [39]. Second, even if we were able to cover a great set of predictors of adherence, some more factors could have been of interest for the research question, such as personality traits or information about social support. Third, the generalisability of these results is limited by inclusion criteria, whereupon only participants with increased risk of dementia were included in the intervention study. Fourth, the intervention took place during the COVID pandemic. Restrictions were reported by the participants especially for enhancement of social activity and physical activity, whereby no effect of perceived restriction on the treatment effect was found [8]. Finally, the sample of AgeWell was quite homogeneous. It is of great interest to extend future interventions to more heterogeneous samples including different groups of ethnicity and minorities.

## Conclusion

This study set out to explore possible predictors, positively and negatively, of adherence to the components of the AgeWell.de multi-domain lifestyle intervention against cognitive decline. The results of this investigation show that better self-efficacy and higher education lead to better adherence, while depressive symptoms and higher BMI-score hindered better adherence as well as smoking. Overall, this study strengthens the idea of more targeted interventions, combined with the enhancement of the adherence to the intervention components by enhancing the self-efficacy of participants as part of the intervention. This paper gives further directions to optimize multi-domain lifestyle interventions.

## Data Availability

The dataset analysed during this study are not publicly available due to privacy restrictions. However, data are available after de-identification to researchers who submit a proposal to the AgeWell.de steering committee (Requests to access the dataset should be directed by correspondence to: Steffi.Riedel-Heller@medizin.uni-leipzig.de).

## Abbreviations

BMI: Body-Mass Index
CAIDE: Cardiovascular Risk Factors, Ageing and Dementia
CASMIN: Comparative Analysis of Social mobility in Industrial Nations scale
FINGER: Finnish Geriatric Intervention Study to Prevent Cognitive Impairment and Disability
GDS: Geriatric Depression Scale
GLM: Generalized Linear Regression Model
GP: General Practitioner
MCI: Mild cognitive impairment
MoCA: Montreal Cognitive Assessment Test
SWE: Scale for General Self-Efficacy
